# Cortical Variability and Challenges for Modeling Approaches

**DOI:** 10.3389/fnsys.2017.00015

**Published:** 2017-04-04

**Authors:** Emili Balaguer-Ballester

**Affiliations:** ^1^Department of Computing and Informatics, Faculty of Science and Technology, Bournemouth UniversityBournemouth, UK; ^2^Bernstein Center for Computational Neuroscience, Medical Faculty Mannheim and Heidelberg UniversityMannheim, Germany

**Keywords:** metastability, excitation-inhibition balance, cortical variability, neuronal variability, recurrent networks, trial to trial variability

The functional role of the observed neuronal variability (the disparity in neural responses across multiple instances of the same experiment) is again receiving close attention in Computational and Systems Neuroscience (e.g., Durstewitz et al., 2010; Moreno-Bote et al., 2011; Oram, 2011; Beck et al., 2012; Churchland and Abbott, 2012; Brunton et al., 2013; Masquelier, 2013; Mattia et al., 2013; Balaguer-Ballester et al., 2014; Renart and Machens, 2014; Bujan et al., 2015; Lin et al., 2015; Pachitariu et al., 2015; Arandia-Romero et al., 2016; Doiron et al., 2016; McDonnell et al., 2016). Special consideration is currently given to understanding how spiking (Bujan et al., 2015; Deneve and Machens, 2016; Doiron et al., 2016; Hartmann et al., 2016; Landau et al., 2016) and phenomenological (Goris et al., 2014; Lin et al., 2015; Mochol et al., 2015; Arandia-Romero et al., 2016; Doiron et al., 2016) models account for the wide range of classical and new phenomena associated with trial-to-trial uncorrelated activity.

Specifically, it has often been proposed that a network state characterized by largely asynchronous spike times whilst maintaining slow oscillations in the firing-rates, may represent the default spontaneous cortical mode (e.g., Sanchez-Vives and Mattia, 2014; Deneve and Machens, 2016; Sancristobal et al., 2016); and similar states could also underlie observed stimulus-driven variability in rate (Litwin-Kumar and Doiron, 2012; Deneve and Machens, 2016; Hartmann et al., 2016). However, the way in which such a computationally advantageous network state for neural coding is achieved can differ substantially between modeling approaches; this challenge will be the focus of this manuscript.

## Predictable components of neuronal variability

The view that the intrinsic stochasticity of single cell activity is the major source of variability has been questioned multiple times over the last decades by modeling (van Vreeswijk and Sompolinsky, 1996; Amit and Brunel, 1997; Shadlen and Newsome, 1998; Deneve et al., 2001; Stein et al., 2005; Faisal et al., 2008; Renart et al., 2010; Rabinovich and Varona, 2011; Masquelier, 2013; Stiefel et al., 2013; Rabinovich et al., 2014; Deneve and Machens, 2016; Hartmann et al., 2016) and empirical studies (e.g., Bryant and Segundo, 1976; Mainen and Sejnowski, 1995; Britten et al., 1996; Stein et al., 2005). It is well known that essentially deterministic networks of balanced excitation and inhibition are able to generate a weakly correlated, often chaotic attractor state which presents Poissonian statistical properties like the observed activity (van Vreeswijk and Sompolinsky, 1996; Amit and Brunel, 1997; Shadlen and Newsome, 1998; Sussillo and Abbott, 2009; Litwin-Kumar and Doiron, 2012). However, such a chaotic state is a non-mandatory modeling choice: recently, a range of models has shown that part of the observed variability may also be explained by a different class of deterministic processes (Beck et al., 2012; Mattia et al., 2013; Renart and Machens, 2014; Bujan et al., 2015; Abbott et al., 2016; Deneve and Machens, 2016; Doiron et al., 2016; Gillary and Niebur, 2016; Hartmann et al., 2016) such as the lack of specificity in top-down processing of cognitively complex tasks (Beck et al., 2012).

At the same time, empirical studies found mounting evidence of deterministic patterns for some of the trial to trial variability. For example, a range of indexes (Shadlen and Newsome, 1998; Churchland and Abbott, 2012; Marcos et al., 2013) suggest that variance is systematically reduced at the stimulus onset (Churchland et al., 2010); and in general shows a predictable trend during different events of the task (Churchland et al., 2006, 2010; Churchland and Abbott, 2012; Ledberg et al., 2012; Renart and Machens, 2014). Thus, there seems to be an increasing consensus in that at least part of the trial to trial variability shows a deterministic pattern which may play a functional role; and hence cannot be simply neglected (Balaguer-Ballester et al., 2011, 2014; Masquelier, 2013; Ecker et al., 2014; Goris et al., 2014; Renart and Machens, 2014; Lin et al., 2015; Schölvinck et al., 2015; Arandia-Romero et al., 2016; Hartmann et al., 2016).

Nevertheless, despite such recent advances, the mapping between the cognitive state and variability is still challenging. For instance, on the one hand, correlated rate variability between pairs of neurons is often reduced by top-down attentional processes (e.g., Cohen and Maunsell, 2009; Mitchell et al., 2009; Cohen and Kohn, 2011; Doiron et al., 2016). On the other hand, the opposite can be observed when attention is highly variable across trials (Roelfsema et al., 2004; Renart and Machens, 2014; Ruff and Cohen, 2014); and such noise correlation analyses show a variety of mixed results (Cohen and Kohn, 2011; Eyherabide and Samengo, 2013; Moreno-Bote et al., 2014; Ruff and Cohen, 2014, 2016; Doiron et al., 2016).

Importantly, compelling evidence suggests that a substantial portion of the spontaneous and evoked total and shared variability is attributable to global fluctuations (Ecker et al., 2014, 2016; Goris et al., 2014; Mochol et al., 2015; Pachitariu et al., 2015; Schölvinck et al., 2015; Arandia-Romero et al., 2016); and this has direct implications in neural coding in visual (Lin et al., 2015; Arandia-Romero et al., 2016; Ecker et al., 2016) and in auditory areas (Mochol et al., 2015; Pachitariu et al., 2015). For instance, high population activity in monkey V1 increases the information that a subset of neuronal ensembles carry about stimulus orientation, only the ones that show a strong multiplicative modulation. In contrast, the stimulus-decoding information of such multiplicative ensembles plummets for low global activity states; whilst information increases in the group additively-modulated neurons in the population (Arandia-Romero et al., 2016).

Global modulations could either stem from the default up/down state of ongoing activity (Mochol et al., 2015) or from fluctuations within a single state (Arandia-Romero et al., 2016). When controlled for this global co-modulations, noise correlations are often negligible (Renart et al., 2010), but not always (Pachitariu et al., 2015; Arandia-Romero et al., 2016). Moreover, stimulus-driven input statistics can also have a strong contribution to the observed evoked variability in parallel to the global network state (Oram, 2011; Bujan et al., 2015; Pachitariu et al., 2015; Doiron et al., 2016; Landau et al., 2016) and explain noise correlations dynamics (Bujan et al., 2015).

This complex variety of results has been recently analyzed using a range of phenomenological and spiking models. These recent modeling efforts aim to pin down when precisely during the course of the trial (Moreno-Bote et al., 2014; Bujan et al., 2015; Doiron et al., 2016) and in which specific network state (Arandia-Romero et al., 2016) noise correlations are informative or deleterious for neural coding (Ecker et al., 2014, 2016; Moreno-Bote et al., 2014; Lin et al., 2015; Pachitariu et al., 2015; Schölvinck et al., 2015; Arandia-Romero et al., 2016; Doiron et al., 2016).

## Diversity of theoretical approaches

The consensus on the network origin of a substantial part of cortical variability led to the development of a multitude of models for explaining the underlying neuronal mechanisms of the asynchronous state (e.g., Boerlin et al., 2013; Deco et al., 2014; Ostojic, 2014; Barral and Reyes, 2016; Hartmann et al., 2016; Rosenbaum et al., 2017). A linking theme in these approaches is the crucial contribution of fast inhibition in recurrent networks; which is negatively correlated with excitation and strong enough to counterbalance it to different degrees (Renart et al., 2010; Deneve and Machens, 2016).

This scenario is currently the subject of a lively debate; and a variety of processing architectures of spiking units have been developed to explain the observed variability phenomena from different perspectives. It has recently been proposed that a much tighter synchronization between excitation and inhibition than considered so far, at the spike level, has an even stronger experimental support and would enable the network to operate optimally by reducing the minimum coding error (Renart et al., 2010; Boerlin et al., 2013; Abbott et al., 2016; Deneve and Machens, 2016). The precise way in which the asynchronous state is achieved however, is not unique. For example, connectivity weights are specifically learnt in the design termed spike-coding network (Boerlin et al., 2013; Schwemmer et al., 2015; Abbott et al., 2016; Deneve and Machens, 2016); whilst connectivity is clustered in Litwin-Kumar and Doiron (2012), shaped by plasticity in Vogels et al. (2011) and Landau et al. (2016) and much less structured in many other dense (Renart et al., 2010; Abbott et al., 2016) or sparse (Ostojic, 2014) networks.

In contrast, other recent approaches rely on non-closely balanced excitatory-inhibitory dynamics in networks of simplified units. For instance, Hartmann et al. (2016) proposed a fully deterministic approach to describe spontaneous and stimulus evoked variability; consisting of an architecture of schematic noise-free units. In this approach, excitatory connectivity is specifically set by plasticity and homeostasis; and is not necessarily balanced. In Deco et al. (2014), the spontaneous state also stems from not necessarily tightly balanced architectures, where elements are field equations derived from spiking units with background input noise. In this and related models, connectivity is also set; but in the latter case local inhibition is regulated by a different homeostatic control. In addition, it has been recently shown that most of the evoked variability could be accounted for by essentially feedforward architectures (Bujan et al., 2015; Doiron et al., 2016).

Asymmetry and slightly unbalanced configurations are also considered to promote the so called metastable state (Mattia et al., 2013; Tognoli and Kelso, 2014a,b; Deco and Kringelbach, 2016), in which high dimensional ensembles flexibly re-organize, synchronize and disengage, possibly by changing their role in a high-dimensional setting (Lapish et al., 2015; Fusi et al., 2016). Such state also exhibits advantageous computational properties (Hellyer et al., 2015; Deco and Kringelbach, 2016). In an instantiation of such ideas, essentially deterministic structures of simplified units generate itinerancy through robust transient states. These states enable the model to process cognitive entities without the compelling need for attractors (Rabinovich et al., 2008, 2014; Varona and Rabinovich, 2016). Moreover, combinations of attractor-based and transient-based computations could also underlie motor plans (Mattia et al., 2013). Other state-dependent computational ingredients such as neuromodulation could also play a major role in shaping the observed variability (Mattia et al., 2013; Lapish et al., 2015; Doiron et al., 2016).

## Future challenges for models

**Figure 1:**
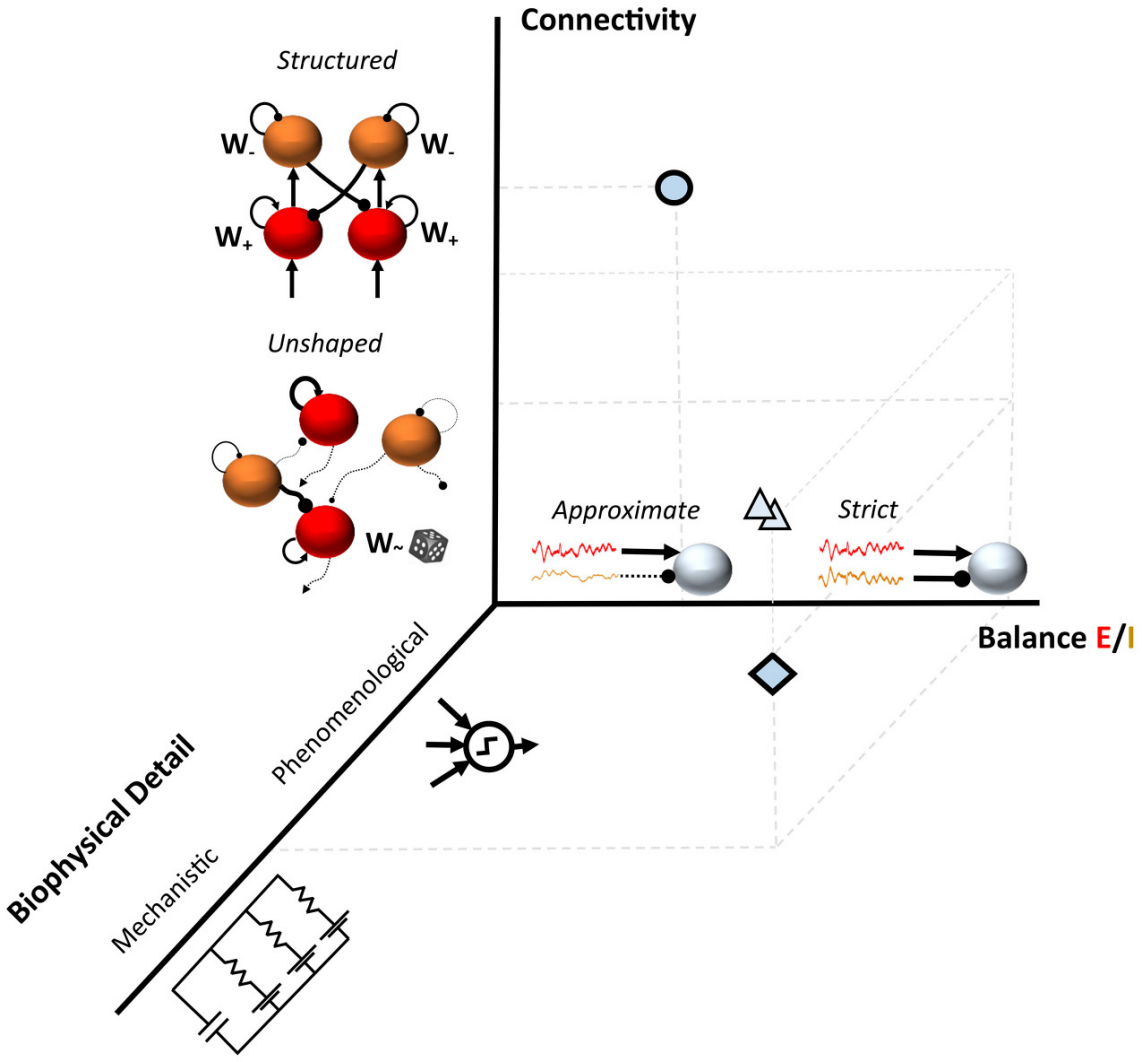
**Three ***dimensions*** in models of neuronal variability**. The diamond shows an example of a spiking model with random connectivity and a tight Excitation/Inhibition (E/I) balance as in Renart et al., 2010; the circle represents a substantially different modeling choice such as in Hartmann et al., 2016. A range of modeling approaches typically fall between these two examples (triangles); such as semi-structured connectivity architectures which modulate the E/I balanced dynamics in realistic networks (Litwin-Kumar and Doiron, 2012; Landau et al., 2016).

This non-exhaustive summary of few recent examples suggests the availability of a plethora of recurrent and feedforward network models for understanding the source of variability during cognitive processing and in the resting state. These configurations often differ at least in the level of detail of the computational units, in the connectivity structure and in the degree of balance between excitation and inhibition (Figure 1).

This challenging scenario perhaps compels to the development of novel approaches for probing the networks in order to identify the suitable architecture or architectures for each specific cognitive process and cortical area. However, the question remains how to effectively dissect a recurrent network, beyond the linearization of the network dynamics, in order to investigate the components originating the asynchronous state (Sussillo, 2014). Recently, Doiron et al. (2016) proposed a framework to identify the physiological processes underlying decorrelation in feedforward circuits by analysing state-dependent correlations in different time windows. However, applying this approach is more problematic in recurrent circuits when coupling is not weak and is highly nonlinear; as is often the case in models.

Hence, inferring which level of detail and architecture are mostly probably responsible for the neuronal variability phenomena is perhaps one of the major challenges for the next years, which possibly requires the development of novel theoretical tools for scrutinizing network behavior.

## Author contributions

The author conceived and wrote the manuscript.

## Funding

This paper has been funded by Bournemouth University. The funders had no role in study design, data collection and analysis, decision to publish, or preparation of the manuscript.

### Conflict of interest statement

The author declares that the research was conducted in the absence of any commercial or financial relationships that could be construed as a potential conflict of interest.
